# Sulfur-Doped BiOCl with Enhanced Light Absorption and Photocatalytic Water Oxidation Activity

**DOI:** 10.3390/nano11092221

**Published:** 2021-08-28

**Authors:** Ruilian Qi, Jian Liu, Huanxiang Yuan, Yu Yu

**Affiliations:** 1 College of Chemistry and Materials Engineering, Beijing Technology and Business University, Beijing 100048, China; qiruilian@btbu.edu.cn; 2 Institute of Chemistry, Chinese Academy of Sciences, Beijing 100090, China; liujian13@iccas.ac.cn; 3 School of Science, Beijing Jiaotong University, Beijing 100044, China

**Keywords:** photocatalytic water oxidation, bismuth oxychloride, doped photocatalyst, density functional theory

## Abstract

Photocatalysis is a powerful strategy to address energy and environmental concerns. Sulfur-doped BiOCl was prepared through a facial hydrothermal method to improve the photocatalytic performance. Experimental results and theoretical calculations demonstrated that the band structure of the sulfur-doped BiOCl was optimally regulated and the light absorption range was expanded. It showed excellent visible-light photocatalytic water oxidation properties with a rate of 141.7 μmol h^−1^ g^−1^ (almost 44 times of that of the commercial BiOCl) with Pt as co-catalyst.

## 1. Introduction

Photocatalysis has been proved to be an effective and promising approach for solar-to-chemical energy conversion and environmental improvement [1,2]. Among the photocatalytic processes, water splitting is an attractive reaction due to the intrinsic cleanliness [3,4,5,6,7,8]. The typical water splitting reaction involves the reduction of protons and oxidation of water molecules, producing hydrogen and oxygen, respectively. However, the sluggish kinetic properties of oxygen evolution greatly limit the solar energy utilization [9,10]. Therefore, it is of great interest to promote the photocatalytic oxygen evolution activity during the water splitting process.

To address the challenge, bismuth oxychloride (BiOCl) has been extensively investigated in the photocatalytic field [11,12]. The unique layered structure of {Cl–Bi–O–Bi–Cl} sheet stacking through nonbonding interactions of BiOCl give it good photocatalytic activities [13]. However, limited light absorption, owing to its wide band gap (ca. 3.5 eV) [14], is the major hurdle for its application. Various methods were employed to extend its light absorption by narrowing the band gap, such as hierarchical nanostructures construction [15,16,17], heterojunction composite design [18,19,20,21,22], and crystal-facet control [23,24,25]. Doping is an effective method to optimize its band gap. The band gap could be significantly adjusted to the visible light region by this method. Halogen mixing [26,27], referring to the replacement of Cl with Br or I atoms, could be achieved with a high doping degree, due to the lower atom exclusiveness and similarity in crystal structure between the bismuth oxyhalide. Doping with nonmetallic (i.e., C [28,29], N [30]) and metallic (i.e., Mn [31,32], Fe [33,34]) elements and particular atomic defects (i.e., oxygen vacancy [35,36,37,38]) were also proved to be able to enhance its photocatalytic activity, by installing an impurity state band to reduce the band gap or providing capturing sites of photo-induced carriers to suppress their recombination. Among them, sulfur doping is an important and effective way to enhance the catalytic activity. Jiang et al. synthesized S-doped BiOCl by a one pot ion liquid-assisted solvothermal method [39]. Shang et al. synthesized sulfur-doped BiOCl by treating BiOCl with hydrochloric acid and thioacetamide [40]. Both the doped BiOCls showed higher activity than before. However, more facile and economic doping strategies are still needed.

We developed a novel strategy to synthesize sulfur-doped BiOCl catalyst (denoted as S-BiOCl) by a simple hydrothermal treatment of commercial BiOCl powder in thiourea solution. S-doped BiOCl displays absorption in the whole UV–vis range, even in the near-infrared region. The narrowed band gap resulted in a great expansion of the light absorption range. In addition, the intrinsic crystal structure of BiOCl and the large interlayer spacing provided induced dipole moments to enhance the separation of light-induced holes and electrons. S-BiOCl loaded with Pt as a co-catalyst showed excellent catalytic activity for visible-light-induced water oxidation.

## 2. Materials and Methods

### 2.1. Materials

Commercial bismuth oxychloride (BiOCl) was purchased from Sigma-Aldrich. Ethanol, thiourea, chloroplatinic acid (H_2_PtCl_6_), sodium borohydride (NaBH_4_), ascorbic acid, and silver nitrate (AgNO_3_) were from Sinopharm Chemical Reagent Co., Ltd. All the reagents were analytically pure and used without further purification. Water used in the experiment was Millipore water prepared by a Milli-Q instrument.

### 2.2. Synthesis of S-BiOCl

One gram of commercial BiOCl powder was added into 20 mL H_2_O and dissolving 0.3 g thiourea. After sufficient stirring and ultrasonic treatment, the mixtures were transferred into a 40 mL Teflon-lined stainless steel autoclave to perform a hydrothermal process at 150 °C for 3 h. After completion of the hydrothermal reaction, the product was washed using ethanol and deionized water thoroughly, then dried at 80 °C in air atmosphere.

### 2.3. Characterization

X-ray diffraction (XRD) patterns were recorded on a Rigaku diffractometer (Maxima XRD-7000) using Cu Ka irradiation. Scanning electron microscope (SEM) images and EDS results were taken with scanning electron microscopy (JEOL-6701F). Transmission electron microscope (TEM) images, EDS results, and SAED were taken with transmission electron microscopy (JEOL-2100F). The UV–visible absorption spectrum of catalysts was obtained by using a UV–visible spectrophotometer (UV-2550, Shimadzu, Japan), and the UV–visible–NIR absorption spectrum was taken with a PerkinElmer Lamda 950. BaSO_4_ was used as a reflectance standard. IR and Raman spectra were obtained on a Nicolet iN10 MX FT-IR microscope. XPS and Raman scattering investigations were carried out on the ESCALab220i-XL photoelectron spectroscope and Thermo Scientific DXR Raman microscope using a 532 nm laser, respectively.

### 2.4. Pt Loading on S-BiOCl

Pt loading was performed according to a previous report with some modifications [41]. One hundred micrograms of dried S-BiOCl powder was added into 500 mL H_2_O with stirring and ultrasonic treatment, and placed in the dark. Then, 0.77 mL 10 mmol/L H_2_PtCl_6_ aqueous solution was added dropwise after stirring for 30 min. Afterwards, 0.1 mL 10 mmol/L NaBH_4_ aqueous solution was dropped, followed by 10 mL H_2_O dissolving 50 mg ascorbic acid 10 min later. The mixtures were stirred overnight, and then washed using absolute ethanol and deionized water several times, and finally dried at 80 °C in air atmosphere. The loading mount of Pt was obtained by dissolving 5 mg loaded catalyst with chloroazotic acid to be measured by ICP.

### 2.5. Photocatalytic Water Oxidation Performance Measurements

Fifty micrograms of Pt-loaded S-BiOCl was ultrasonically dispersed in 100 mL 0.01 mol/L AgNO_3_ aqueous solution, and then transferred into a quartz container of the Perfect Light Labsolar-IIIAG water splitting system (Figure S1). After reaching the vacuum requirements, it was exposed to visible light irradiation by a 200 W Xe lamp with a 420 and 780 nm cut-off filter under continuous stirring. A small quantity of gas was sampled at 30 min or 1 h to measure the oxygen yield. Quantitative determination of oxygen gas was conducted on a GC7980 gas chromatograph with the 5A molecular sieve type capillary column (2 mm × 3 mm × 2 m stainless steel, No. 6350101) and a TCD detector. The area percentage method based on the prepared standard curve was adopted in quantification.

### 2.6. Theoretical Calculation

All calculations were carried out based on density functional theory (hybrid functional, HSE06) using the Vienna ab initio simulation package (VASP) [42]. The generalized gradient approximation (GGA) using the Perdew–Burke–Ernzerhof (PBE) exchange correlation functional was employed, with cut-off energy of 500 eV, total energy convergence of 1 × 10^−4^ eV, and force convergence of 0.01 eV Å^−1^. In view of the doping ratio of sulfide, a 3 × 3 × 1 supercell was constructed to represent BiOCl (Figure S5a). Three doping models in which two S atoms were located at different sites were considered (denoted as BiOCl_2/3_S_1/3_, Figure S5d,g,j). The 2s and 2p states were considered for O with 6 electrons, as 3s and 3p states were adopted for Cl and S with 7 and 6 electrons, respectively. For Bi, besides 6s and 6p, the semicore 5d states were also included with a total of 15 electrons. The Monkhorst−Pack scheme Kpoints grid sampling was set as 6 × 6 × 3 for the irreducible Brillouin zone. The band structures were calculated along the paths connecting the following high symmetry points: Z(0,0,0.5), A(0.5,0.5,0.5), M(0.5,0.5,0), Γ(0,0,0), Z(0,0,0.5), R(0,0.5,0.5), X(0,0.5,0), Γ(0,0,0) in the k-space [14].

## 3. Results and Discussion

### 3.1. Preparation and Characterizations of S-BiOCl

**Figure 1:**
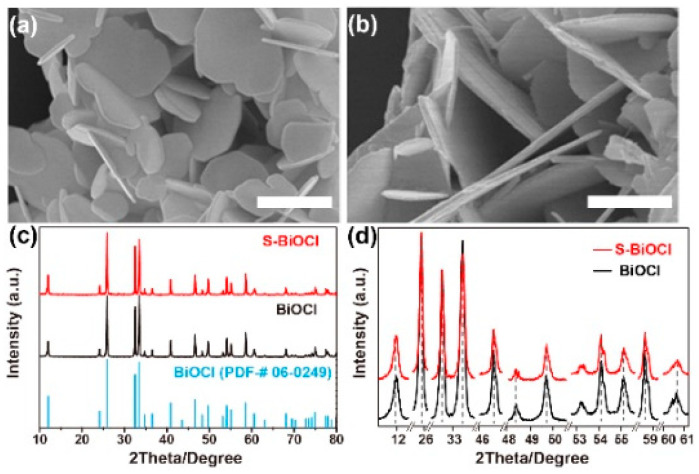
SEM images of (**a**) commercial BiOCl and (**b**) S-BiOCl. Scale bar: 1 μm. (**c**) XRD patterns and (**d**) partial amplified XRD patterns of commercial BiOCl and S-BiOCl.

S-BiOCl was prepared by a facile hydrothermal treatment of commercial BiOCl powder in thiourea solution. The morphology of S-BiOCl was characterized by SEM and TEM. As shown in Figure 1, the commercial BiOCl and S-BiOCl both exhibited homogeneous sheet-like structures. Compared to commercial BiOCl, no obvious aggregating, collapsing, or morphological changes were observed on S-BiOCl sheets. Additionally, no particles or other structures were observed attached on the surface of the sheets. XRD analysis in Figure 1c revealed the preservation of tetragonal BiOCl phase (PDF#06-0249), while neither sulfide nor sulphochloride was detected, indicating that only doping happened during the hydrothermal reaction rather than dissolving, recrystallization, or growth. Furthermore, no peak shift representing the change in the lattice parameter was observed in partial amplified XRD patterns of commercial BiOCl and S-BiOCl, indicating the absence of appreciable lattice distortion (Figure 1d).

**Figure 2:**
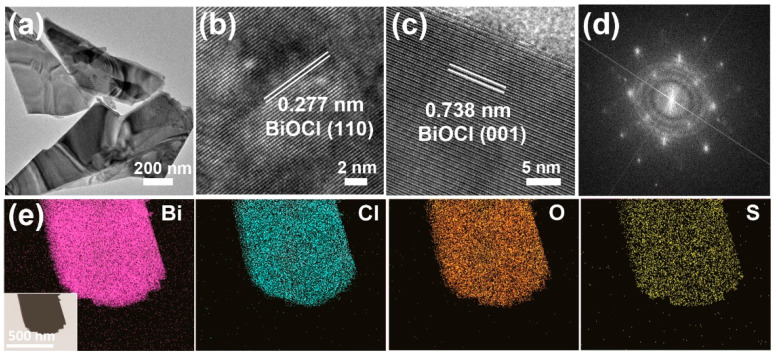
(**a**) TEM, (**b**) top-view HRTEM, (**c**) side-view HRTEM, (**d**) SAED of S-BiOCl, and (**e**) STEM-EDS elemental mapping of Bi, Cl, O, and S of S-BiOCl sheet (inset of (**e**) is STEM image of the corresponding area).

The TEM image of S-BiOCl (Figure 2a) further confirmed the uniformity and homogeneity of the sheets. The high-resolution TEM (HRTEM) images (Figure 2b,c) showed uniform fringe spaces of 0.277 nm, due to (110) lattices of the tetragonal BiOCl, while the interlayer spacing of 0.738 nm corresponding to (001) lattice fringes was observed in the side-view image. The SAED image (Figure 2d) demonstrated the single crystallinity of S-BiOCl sheets. In the elemental mapping of S-BiOCl (Figure 2e), all the four elements were uniformly distributed through the whole sheet, indicating that S was homogeneously doped in the sheet. The analysis of element composition by EDS, together with the XPS, is shown in Table 1. The quantitative decrease in Cl, compared to the stoichiometric ratio of Cl in BiOCl, was very close to the proportion of doped S, which implied that BiOCl was doped in the form of the substitution of Cl atoms with S atoms.

**Figure 3:**
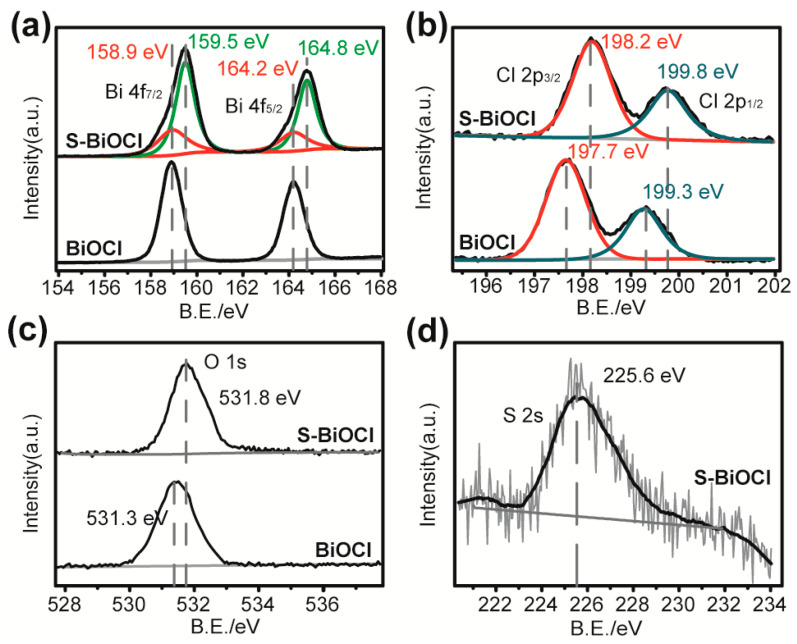
(**a**) Bi 4f XPS spectra, (**b**) Cl 2p XPS spectra, and (**c**) O 1s XPS spectra of S-BiOCl and commercial BiOCl. (**d**) S 2s XPS spectra of S-BiOCl.

The XPS survey showed the variation in the valence state of S-BiOCl after doping (Figure 3a–c). In detail, the Bi 4f_7/2_ and 4f_5/2_ peaks were more constitutive of the main ones at 159.5 and 164.8 eV, which positively shifted by 0.6 eV, than the common peaks of Bi^3+^ in commercial BiOCl [43]. Similar changes also happened to Cl 2p and O 1s bands, both of which integrally shifted to high bonding energy by 0.5 eV [44,45]. The above observations suggested that Bi, O, and Cl in doped samples were partially or totally equipped with more positive valencies than conventional +3, −2, and −1. The valent characteristics of doped S, whose 2s band is located at 225.6 eV in Figure 3d, are very close to the −2 state [46,47] and dissimilar to characteristic peak of S 2s in thiourea, which was at 228.6 eV [48]. Meanwhile, no detectable N signal was found (Figure S2), indicating that no thiourea or its derivatives were left in S-BiOCl. Combined with the element composition data by different techniques and the absence of Bi_2_S_3_ or BiSCl depicted by XRD, it was fair to conclude that the S element in the valency of −2 was interpolated into the crystal structure to replace the Cl^−^ anions. Meanwhile, the negative valency of S^2−^ relative to Cl^−^ was balanced by the increasing of binding energy and valency of other three elements. The doping of the S element and the resulting effect on the crystal structure were also reflected in the IR and Raman spectra of S-BiOCl (Figure S3). A new band appeared at 1120 cm^−1^ in IR, which was very close to the characteristic 1106 cm^−1^ band from Bi-S vibration in Bi_2_S_3_ [44]. In the Raman spectra, the peak at 100 cm^−1^ and the broad band (200–300 cm^−1^) with the maximums near 240 and 267 cm^−1^ were similar to the strongest A_g_ and B_1g_ Bi-S stretching bands of Bi_2_S_3_ [44,49].

The above results confirmed the successful doping of sulfur into the commercial BiOCl. Unlike doping from the BiOCl precursors, the doping procedure carried out with crystallized BiOCl enabled the sulfur species to selectively attack the Bi-Cl dangling bonds, which have less bond energy in the interlayer space, rather than the Bi-O with greater bond energy inside the compact and robust [Bi_2_O_2_]^2+^ layers. The large interlayer spacing, as much as 0.738 nm, allowed sulfur species to infiltrate and replace the Cl atoms exposed outside of the [Bi_2_O_2_]^2+^ layers. Furthermore, the ionic radius of S^2−^ (0.184 nm) was nearly equal to that of Cl^−^ (0.181 nm), and much bigger than O^2−^ (0.140 nm) [50]. For S^2−^, the substitution for the Cl^−^ of a similar size in a comparatively loose lattice environment may cause smaller lattice distortions in the crystal structure, resulting in smaller increases in system energy. XPS indicated that there were no positive valent S components in doped samples, which meant that no substitution for Bi^3+^ with S^2+^ or S^4+^ occurred. The dimensional similarity and the resulting doping under the preponderant maintenance of the crystal structure offered the possibility of a doping degree as high as 30% (calculated from the XPS results) in this work. This doping degree was similar to the halogen mixing and much higher than conventional sulfur doping in TiO_2_ and others [51].

### 3.2. Band Structure and Photocatalytic Property of BS-CN

**Figure 4:**
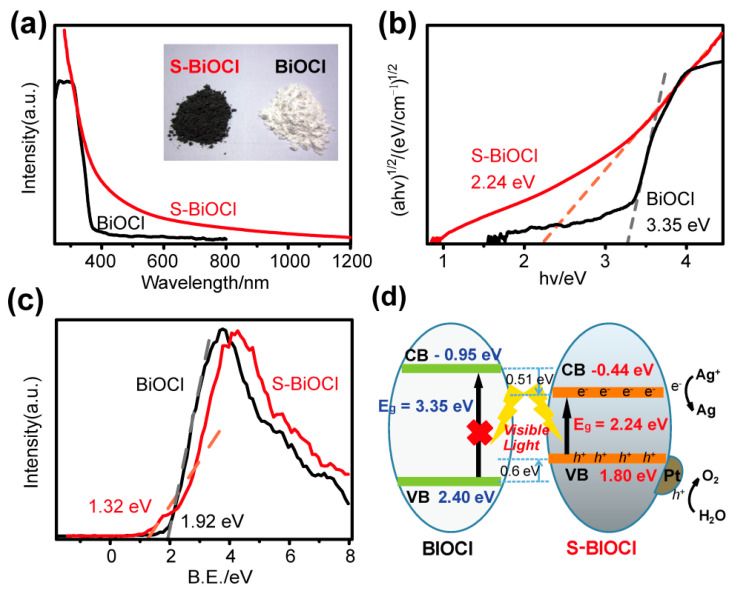
(**a**) UV–vis–NIR absorption spectra (inset is the optical image), (**b**) band gap fitted values by Kubelka–Munk function, (**c**) valence band XPS spectra, and (**d**) the illustration of band structures of commercial BiOCl and S-BiOCl.

Although the crystal structure of S-BiOCl was maintained after doping, its optical property changed significantly. Unlike commercial BiOCl, in the UV–vis–NIR absorption spectra of Figure 4a, S-BiOCl exhibited a continuous absorption in the full range of 400–800 nm, even extending to 1000 nm. The inset of Figure 4a showed that the commercial BiOCl is a white powder, while the S-BiOCl showed a much darker color. The difference in atomic and electronic structures between S and Cl atoms had a substantial effect on the atomic valence states and electronic structure of S-BiOCl. The band gap of S-BiOCl estimated by the Kubelka–Munk function was 2.24 eV (Figure 4b), much less than that of pristine BiOCl (3.35 eV). Such a small band gap tremendously enhanced the light absorption efficiency of S-BiOCl.

In addition to the decrease in the band gap, as the XPS valence band (VB) spectrum showed in Figure 4c, the VB of commercial BiOCl displayed an energy absorption edge at about 1.92 eV, while that of S-BiOCl upshifted to 1.32 eV. Based on the fact that the valence band maximum (VBM) of pure BiOCl was at 2.40 eV [52] (vs. NHE), through theoretical computation, the VBM of S-BiOCl was calculated to be at 1.80 eV. According to the optical absorption spectrum and band gap value estimated by the Kubelka–Munk function, the conduct band minimum (CBM) of S-BiOCl would occur at −0.44 eV, downshifted by 0.51 eV relative to that of pure BiOCl at −0.95 eV (Figure 4d).

**Figure 5:**
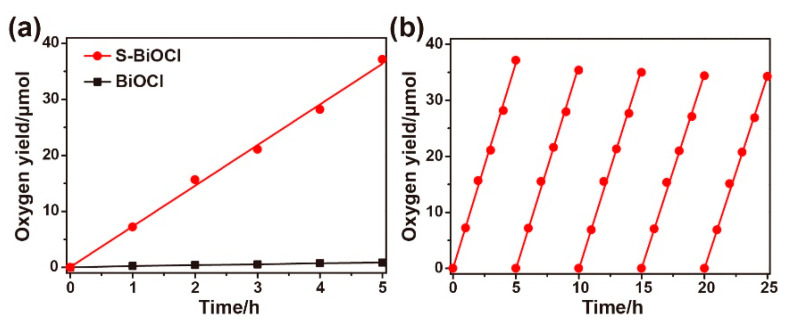
(**a**) The photocatalytic O_2_ evolution activities of S-BiOCl and commercial BiOCl. (**b**) The cycling test of S-BiOCl.

The photocatalytic water oxidation performance of S-BiOCl was studied, with loaded Pt nanoparticles as co-catalyst and silver ions as a sacrificial agent. Pt particles at about 5 nm were successfully loaded on the surface of S-BiOCl sheets by the synergetic reduction of NaBH_4_ and ascorbic acid in the dark. The inset of Figure S4d clearly shows Pt (111) lattice fringes of 0.227 nm and BiOCl (103) lattice fringes of 0.206 nm. The loading amount of Pt measured by ICP was 1.15 wt%. The Pt-loading S-BiOCl exhibited good and stable oxygen production efficiency in 0.01 mol/L AgNO_3_ aqueous solution under visible radiation. As shown in Figure 5a, 37.1 μmol O_2_ was generated after photoirradiation for 5 h when S-BiOCl was used as the catalyst, while only 0.85 μmol O_2_ was detected with commercial BiOCl as the catalyst. The rate of oxygen generation of S-BiOCl was 141.7 μmol h^−1^ g^−1^, which was almost 44 times of that of the commercial BiOCl. Compared with the recent literature (Table S1), S-BiOCl also showed excellent photocatalytic oxygen evolution activity. Moreover, the S-BiOCl showed satisfactory stability in a 25 h cyclic test (Figure 5b).

### 3.3. DFT Calculations

**Figure 6:**
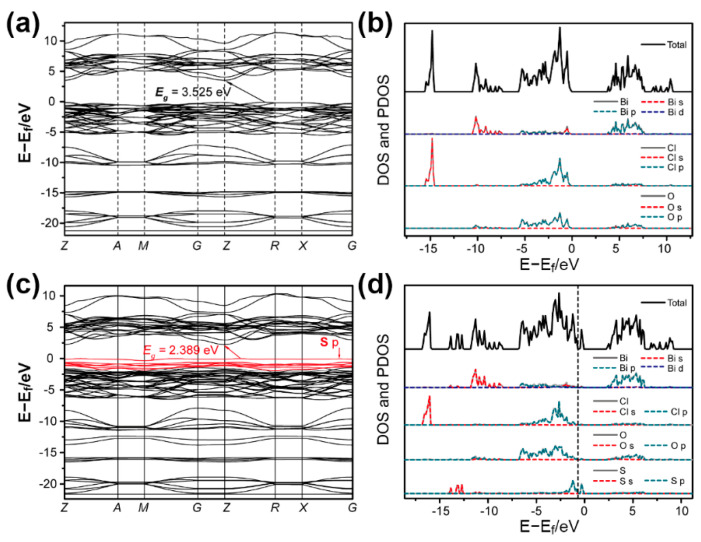
(**a**,**c**) Calculated band structures, and (**b**,**d**) calculated total and partial density of states of BiOCl (**a**,**b**) and BiOCl_2/3_S_1/3_ (**c**,**d**).

To understand the relationship between the structure and the excellent photocatalytic performance in depth, the theoretical calculation based on density functional theory (hybrid functional, HSE06) using the Vienna ab initio simulation package [42] was employed to investigate the variation in the band structure after S doping. Three models (Figure S5d,g,j) were considered according to the S content (almost 30% Cl was substituted) and stable configurations. Compared to the BiOCl (Figure 6a, E_g_ = 3.525), S doping caused an S 2p state as the VBM, and narrowed the E_g_ of S-BiOCl to 2.389 eV (Figure 6c). Although the decrease in E_g_ caused the downshift of CBM and the upshift of VBM, its VBM was still much more positive than E_0_(O_2_/H_2_O) (1.229 eV vs. NHE) [53]. The total density of state (DOS) and partial density of state (PDOS) results of BiOCl and S-BiOCl (Figure 6b,d) also showed that the emergence of a new energy state in the forbidden band of BiOCl was mainly attributed to the hybridization of S 2p with a little of Bi 6s and O 2p. Furthermore, Bi 6p could primarily account for the hybridization of a more dispersed CB, giving rise to the downshift of CBM. Three doping models in which two S atoms were located at different sites were calculated (Figure S5d–l). All the models gave a narrowed E_g_ from 1.672–2.389 eV (Figure S6). The calculation results indicated that S doping can narrow the E_g_ of BiOCl, which can enhance the light harvesting of the catalyst. In addition, S doping could result in higher mobility of photoinduced electrons in CB and holes in VB and inhibition of their recombination [54]. Hence, S-BiOCl exhibits much better oxygen evolution performance than commercial BiOCl.

## 4. Conclusions

In summary, we developed a facile strategy to prepare sulfur-doped BiOCl catalyst through hydrothermal treatment of commercial BiOCl powder in thiourea solution. The S-BiOCl displayed absorption in the full range of the UV–vis region. Various characterizations were employed to prove that S atoms substituted Cl atoms in the dihalide layer. Due to the high doping level and the difference between S and Cl atoms, the electronic and band structure of S-BiOCl changed to favor a narrowed band gap, which was verified by the theoretical calculation. The maintained large interlayer spacing enhanced the separation of light-induced holes and electrons. S-BiOCl, with loaded Pt as co-catalyst, showed good and stable catalytic activity for visible-light-induced water oxidation.

## Figures and Tables

**Table 1:** Elemental composition of S-BiOCl catalyst (at. %) by EDS-SEM, EDS-TEM, and XPS.

Bi	O	Cl	S	Method
30.7	38.8	25.3	5.2	EDS-SEM
32.1	33.2	26.2	8.5	EDS-TEM
29.0	38.8	22.7	9.5	XPS

## Data Availability

The data presented in this study are available on request from the corresponding author.

## Supplementary Materials

The following are available online at https://www.mdpi.com/article/10.3390/nano11092221/s1, Figure S1: Picture of the Perfect Light Labsolar-IIIAG water splitting system; Figure S2: (a) XPS survey, (b) C 1s, and (c) N 1s spectra of S-BiOCl. C 1s spectrum of pure BiOCl is also displayed in (b); Figure S3: (a) IR and (b) Raman spectra of commercial BiOCl and S-BiOCl. IR spectrum of thiourea is also displayed; Figure S4: (a) low- and (b) high-magnification SEM, and (c) low- and (b) high-magnification TEM images of Pt-loaded S-BiOCl. HRTEM image is presented in the inset of (d); Figure S5: (a, d, g, j) Crystal structure models, (b, e, h, k) calculated band structures, and (c, f, i, l) calculated total and partial density of states of BiOCl (a–c) and three kinds of BiOCl_2/3_S_1/3_ (d–l); Figure S6: The schematic diagrams of calculated band structures of pure and S-doped BiOCl; Table S1. Summary of the catalytic oxygen evolution activity of bismuthum-based catalysts.
