# Supplementary material for: Sulfur-Doped BiOCl with Enhanced Light Absorption and Photocatalytic Water Oxidation Activity
Source: Nanomaterials (Basel). 2021 Aug 28;11(9):2221. doi: 10.3390/nano11092221 (PMC8472310; doi:10.3390/nano11092221)
Supplement: Supplementary file 1 [file nanomaterials-11-02221-s001.zip › nanomaterials-1354314-supplementary.pdf]

nanomaterials

MDPI

Supplementary material

Sulfur-Doped BiOCl with Enhanced Light Absorption and Photocatalytic Water Oxidation Activity

Ruilian Qi [1], Jian Liu [2], Huanxiang Yuan [1,*] and Yu Yu [3,*]

[1] College of Chemistry and Materials Engineering, Beijing Technology and Business University. Beijing 100048, China; qiruilian@btbu.edu.cn
[2] Institute of Chemistry, Chinese Academy of Sciences; Beijing 100090, China; liujian13@iccas.ac.cn
[3] School of Science, Beijing Jiaotong University. Beijing, 100044, China; yuyu@bjtu.edu.cn
* Correspondence: yhx@iccas.ac.cn (H. Y.); yuyu@bjtu.edu.cn (Y. Y.)

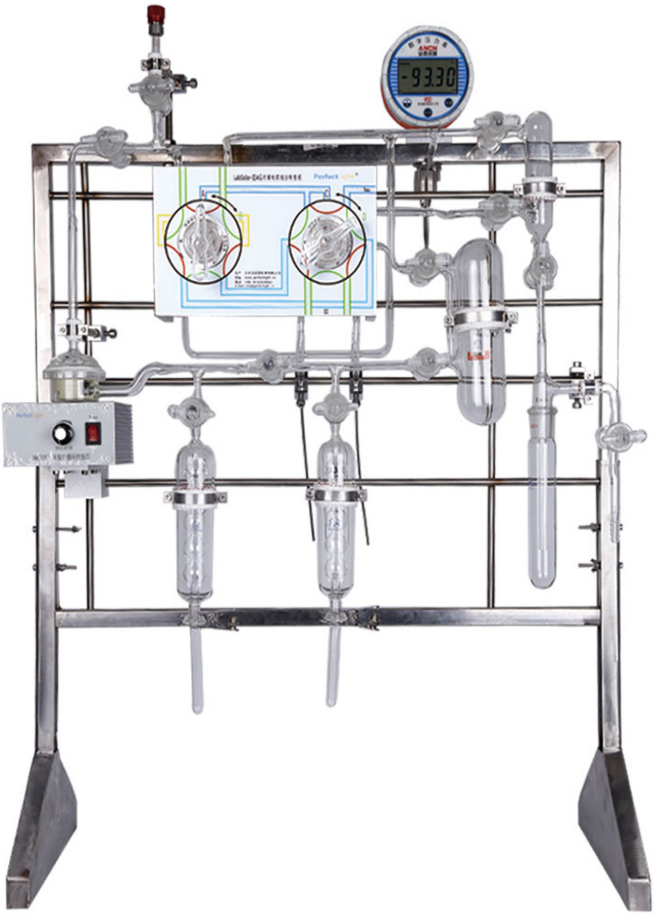

Figure S1. Picture of the Perfect Light Labsolar-IIIAG water splitting system.

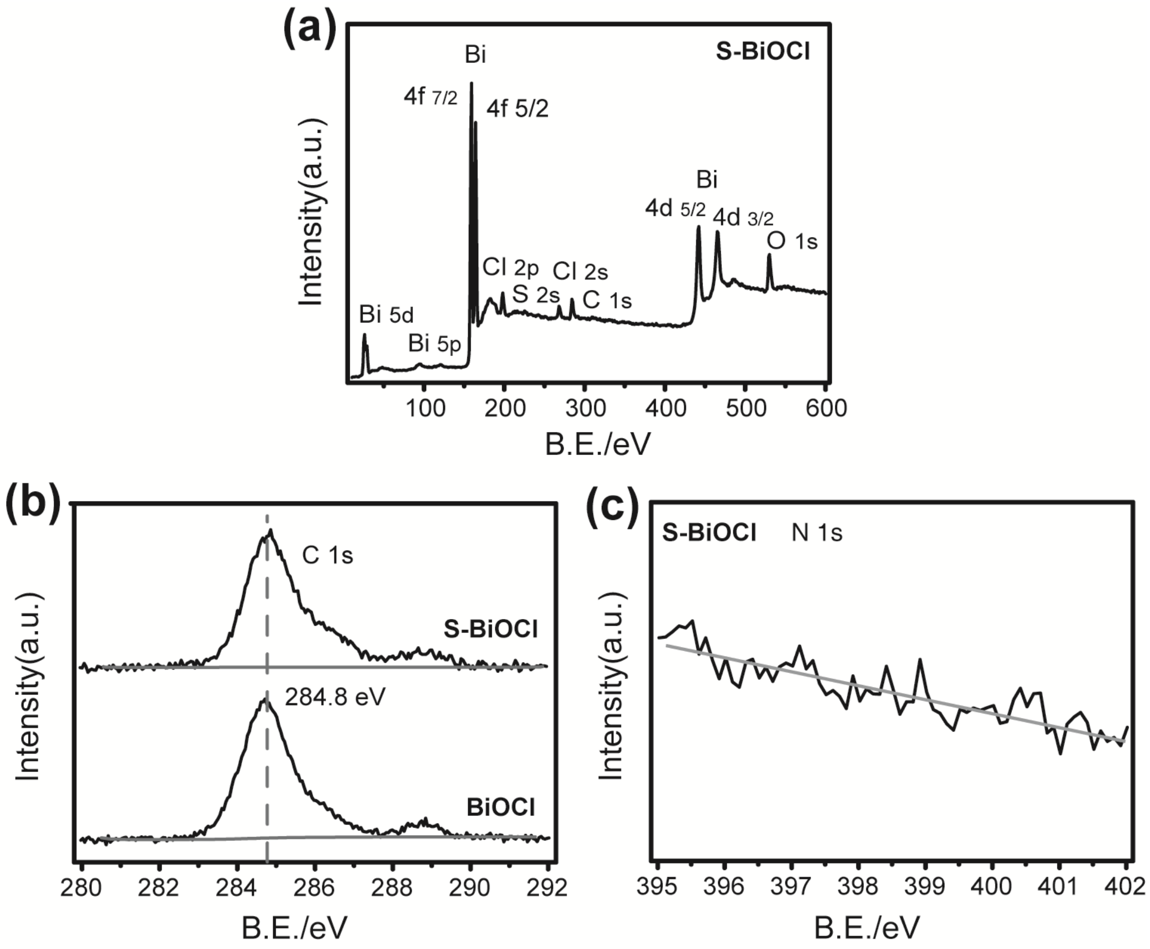

Figure S2. (a) XPS survey, (b) C 1s, and (c) N 1s spectra of S-BiOCl. C 1s spectrum of pure BiOCl is also displayed in (b).

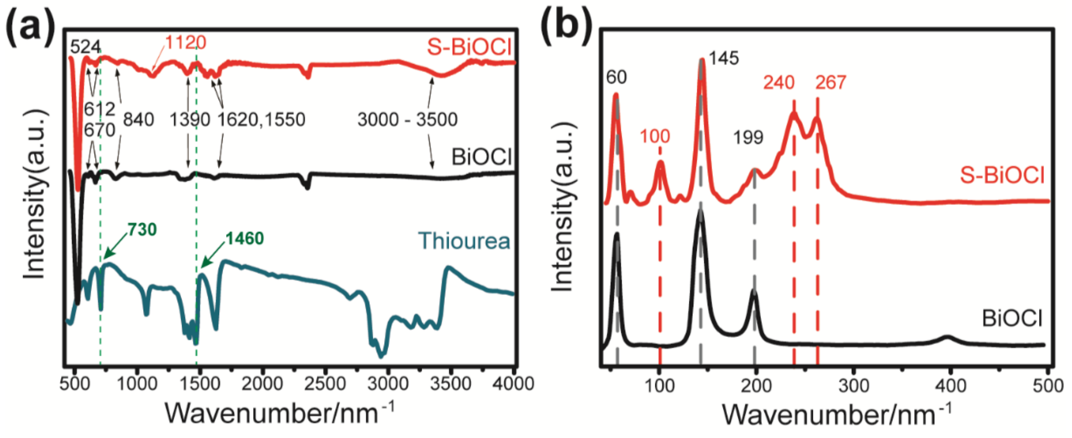

Figure S3. (a) IR and (b) Raman spectra of commercial BiOCl and S-BiOCl. IR spectrum of thiourea is also displayed.

In IR analysis, S-BiOCl retained most characteristic peaks of commercial BiOCl, including the strongest peak at 524 cm^{-1} and others at 612, 670, and 840 cm^{-1} assigned to the symmetrical stretching of Bi-O in the Bi-O_3 pyramid of $[Bi_2O_2]^{2+}$ layers. The feature at 1390 cm^{-1} was associated with the bonding of Bi atoms on the outer edge of the $[Bi_2O_2]^{2+}$ layers with Cl atoms in the bi-halide layer [1, 2]. The new band at 1120 cm^{-1} was much closer to

the characteristic at 1106 cm^{-1} caused by Bi-S vibration in Bi_2S_3 [3], which indicated the probable presence of similar Bi-S bonding in S-BiOCl. We also investigated the IR properties of thiourea, which displayed an apparent peak at 1080 cm^{-1}. However, this band had a comparatively larger deviation from the 1106 cm^{-1} band in S-BiOCl, and it is hard to say whether the latter could be ascribed to NH_2 rocking modes in residual thiourea. Furthermore, the distinct characteristics of thiourea at 730 and 1460 cm^{-1} and the series ranging from 2600 to 3500 cm^{-1} were not detected [4], indicating that thiourea was completely cleared away by washing. It was also confirmed that the feature at 1390 cm^{-1} indeed derived from the Bi-Cl bond. Additionally, the hydrothermal treatment produced plenty of hydroxyl on the surface of S-BiOCl, which gave intensive adsorption at 1550 and 1620 cm^{-1} and in the range of 3000–3500 cm^{-1} [2, 3].

In Raman spectra, the characteristic peaks at 60, 145, and 199 cm^{-1}, representing the A_{1g} external, A_{1g} internal, and E_g internal stretching mode of BiOCl, were observed in both commercial and S-doped BiOCl [3, 5]. The new and apparent peak at 100 cm^{-1} and broad and continuous band (200–300 cm^{-1}) appeared with the maximums near 240 and 267 cm^{-1} of S-BiOCl, which coincided well with the strongest A_g and B_{1g} Bi-S stretching bands of Bi_2S_3 [3, 6]. Both IR and Raman analysis showed that some similar Bi-S bonding produced by the S doping existed in S-BiOCl.

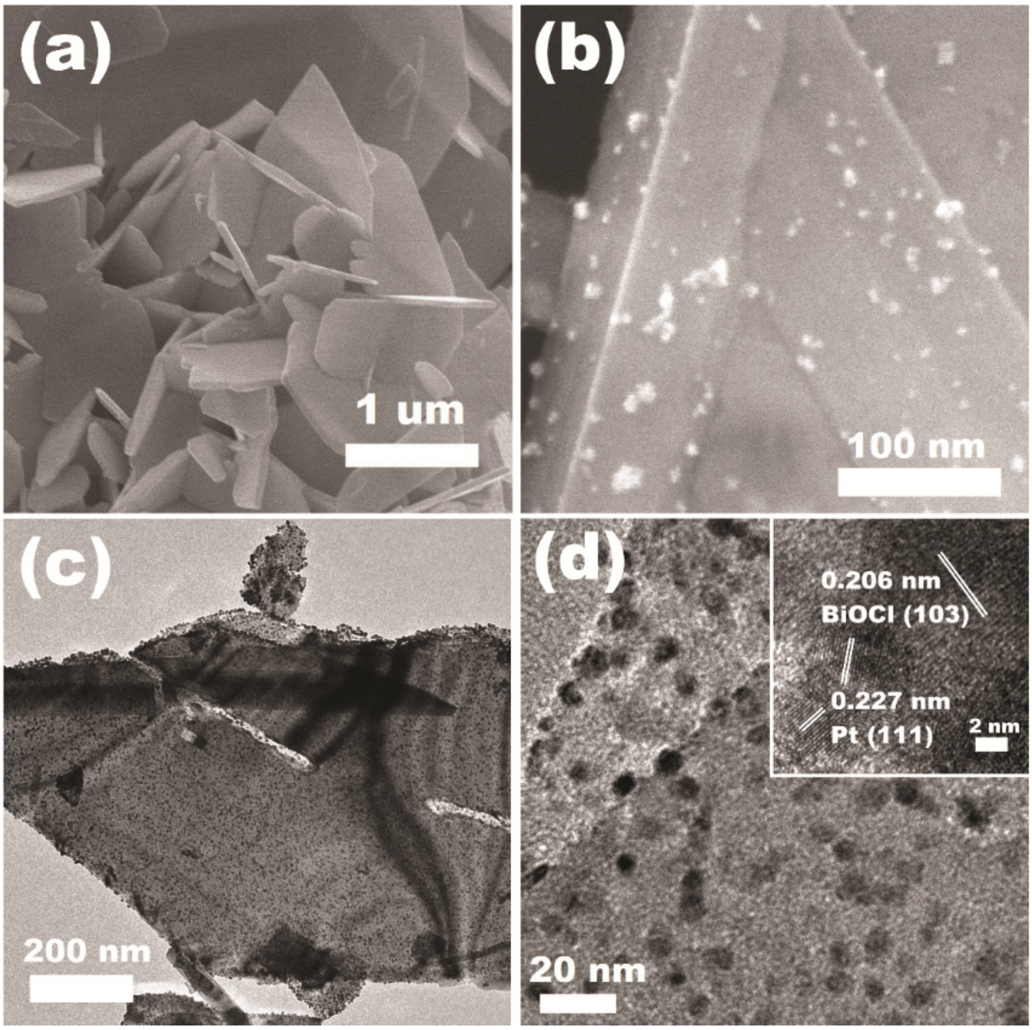

Figure S4. (a) Low- and (b) high-magnification SEM, and (c) low- and (b) high-magnification TEM images of Pt-loaded S-BiOCl. HRTEM image is presented in the inset of (d).

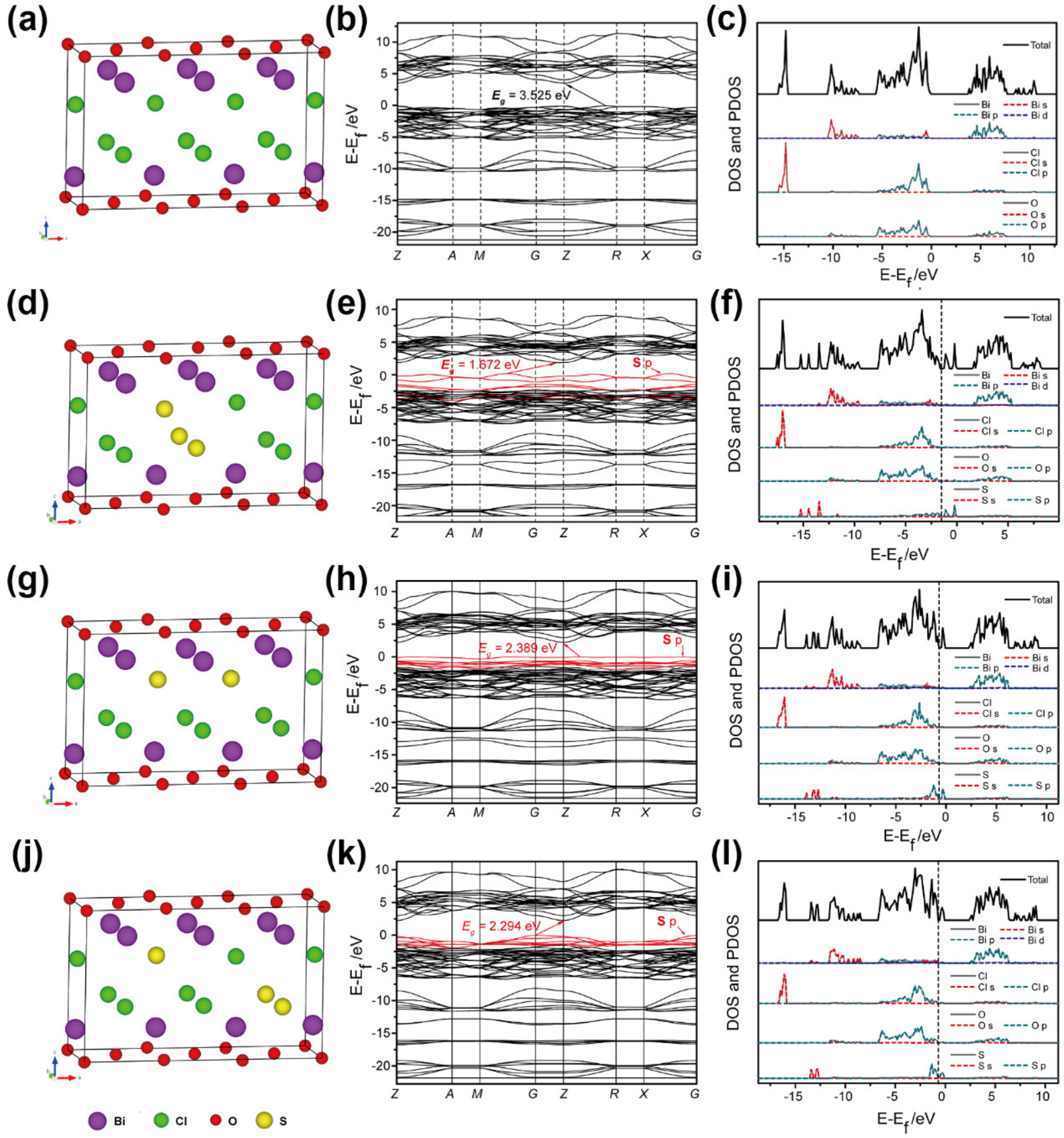

Figure S5. (a, d, g, j) Crystal structure models, (b, e, h, k) calculated band structures, and (c, f, i, l) calculated total and partial density of states of BiOCl (a-c) and three kinds of $BiOCl_{2/3}S_{1/3}$ (d-l).

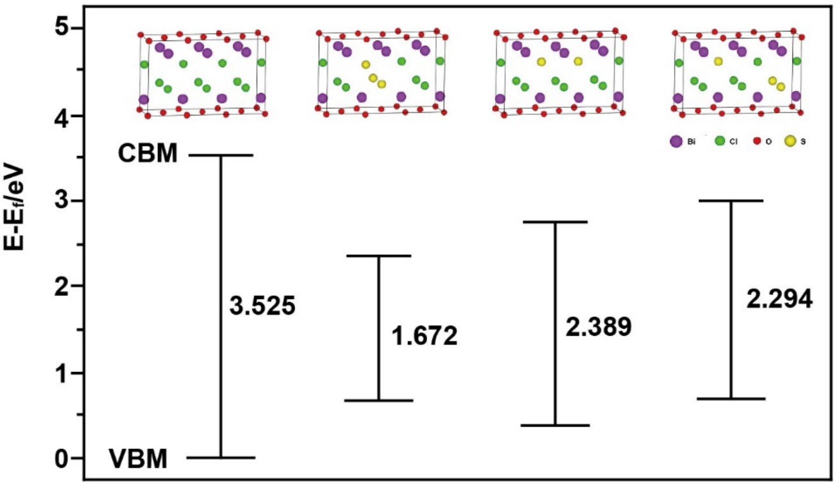

Figure S6. The schematic diagrams of calculated band structures of pure and S-doped BiOCl.

Table S1. Summary of the catalytic oxygen evolution activity of bismuthum-based catalysts.

Photocatalyst	Light source	Reaction conditions	O_2 evolution rate (μmol g^{-1} h^{-1})	Ref.
S-doped BiOCl	200 W Xe lamp, 780 nm > λ > 420 nm	100 mL H_2O, $AgNO_3$ (0.01 M) Pt cocatalyst	141.7	This work
2D–2D Bi3O4Cl/BiOCl	300 W Xe lamp, λ > 400 nm	$AgNO_3$ and $FeCl_3$ (0.05 M)	58.6	[7]
Bi2WO6 nanosheets	300 W Xe lamp, λ > 420 nm	100 mL H_2O, $AgNO_3$ (0.05 M)	100.13	[8]
BP/BiOBr	300 W Xe lamp, 780 nm > λ > 420 nm	100 mL H_2O, $AgNO_3$ (1 g)	89.5	[9]
BP/BiVO4	300 W Xe lamp, (> 420 nm)	$AgNO_3$ solution	102	[10]
Ag3PO4/h-BN-2	400 W Xe lamp, (> 420 nm)	$AgNO_3$ solution	48	[11]
BiVO4/rGO	300 W Xe lamp, (> 420 nm)	$AgNO_3$ solution	107	[12]

References

1. Ye, L.; Gong, C.; Liu, J.; Tian, L.; Peng, T.; Deng, K.; Zan, L., Bin(Tu)xCl3n: a novel sensitizer and its enhancement of BiOCl nanosheets' photocatalytic activity. *Journal of Materials Chemistry* **2012,** *22* (17), 8354-8360.
2. Cheng, G.; Xiong, J.; Stadler, F. J., Facile template-free and fast refluxing synthesis of 3D desertrose-like BiOCl nanoarchitectures with superior photocatalytic activity. *New J. Chem.* **2013,** *37* (10), 3207-3213.
3. Cao, J.; Xu, B.; Lin, H.; Luo, B.; Chen, S., Novel Bi_2S_3-sensitized BiOCl with highly visible light photocatalytic activity for the removal of rhodamine B. *Catal. Commun.* **2012,** *26*, 204-208.
4. Stewart, J. E., Infrared Absorption Spectra of Urea, Thiourea, and Some Thiourea-Alkali Halide Complexes. *J. Chem. Phys.* **1957,** *26* (2), 248-254.
5. Weng, S.; Chen, B.; Xie, L.; Zheng, Z.; Liu, P., Facile in situ synthesis of a Bi/BiOCl nanocomposite with high photocatalytic activity. *J. Mater. Chem. A* **2013,** *1* (9), 3068-3075; Davies, J. E. D., Solid state vibrational spectroscopy—III[1] The infrared and raman spectra of the bismuth(III) oxide halides. *Journal of Inorganic and Nuclear Chemistry* **1973,** *35* (5), 1531-1534.
6. Zhao, Y.; Chua, K. T. E.; Gan, C. K.; Zhang, J.; Peng, B.; Peng, Z.; Xiong, Q., Phonons in Bi 2 S 3 nanostructures: Raman scattering and first-principles studies. *Physical Review B* **2011,** *84* (20), 205330.
7. Ning, S.; Shi, X.; Zhang, H.; Lin, H.; Zhang, Z.; Long, J.; Li, Y.; Wang, X., Reconstructing Dual-Induced {0 0 1} Facets Bismuth Oxychloride Nanosheets Heterostructures: An Effective Strategy to Promote Photocatalytic Oxygen Evolution. *Solar RRL* **2019,** *3* (5), 1900059.
8. Di, J.; Chen, C.; Zhu, C.; Ji, M.; Xia, J.; Yan, C.; Hao, W.; Li, S.; Li, H.; Liu, Z., Bismuth vacancy mediated single unit cell Bi2WO6 nanosheets for boosting photocatalytic oxygen evolution. *Applied Catalysis B: Environmental* **2018,** *238*, 119-125.
9. Li, X.; Xiong, J.; Gao, X.; Ma, J.; Chen, Z.; Kang, B.; Liu, J.; Li, H.; Feng, Z.; Huang, J., Novel BP/BiOBr S-scheme nano-heterojunction for enhanced visible-light photocatalytic tetracycline removal and oxygen evolution activity. *J. Hazard. Mater.* **2020,** *387*, 121690.
10. Zhu, M.; Sun, Z.; Fujitsuka, M.; Majima, T., Z-Scheme Photocatalytic Water Splitting on a 2D Heterostructure of Black Phosphorus/Bismuth Vanadate Using Visible Light. *Angew. Chem. Int. Ed. Engl.* **2018,** *57* (8), 2160-2164.
11. Liu, H.; Wang, X.; Lan, Z., Cryogenic ball milling synthesis of Ag3PO4/h-BN nanoparticles with increased performance for photocatalytic oxygen evolution reaction. *Ceram. Int.* **2019,** *45* (13), 16682-16687.
12. Xie, Z.; Tan, H. L.; Wen, X.; Suzuki, Y.; Iwase, A.; Kudo, A.; Amal, R.; Scott, J.; Ng, Y. H., The Importance of the Interfacial Contact: Is Reduced Graphene Oxide Always an Enhancer in Photo(Electro)Catalytic Water Oxidation *ACS Appl Mater Interfaces* **2019,** *11* (26), 23125-23134.
